# A nutrient-dependent division antagonist is regulated post-translationally by the Clp proteases in *Bacillus subtilis*

**DOI:** 10.1186/s12866-018-1155-2

**Published:** 2018-04-06

**Authors:** Norbert S. Hill, Jason D. Zuke, P. J. Buske, An-Chun Chien, Petra Anne Levin

**Affiliations:** 1 Department of Biology, Washington University, St. Louis, 63130 MO USA; 2 Present address: Department of Molecular and Cell Biology, University of California, Berkeley, 94720 CA USA; 3 Present address: Department of Bacteriology, University of Wisconsin, Madison, 53706 WI USA; 4 Present address: Clinical Immunology and Bioanalysis, MedImmune LLC, South San Francisco, 94080 CA USA; 5 Leukaemia & Blood Cancer Research Unit, University of Auckland, Private Bag 92019, Auckland, 1142 New Zealand

**Keywords:** Cell cycle, Cell size, Cell division, ClpP, UgtP, UDP-glucose

## Abstract

**Background:**

Changes in nutrient availability have dramatic and well-defined impacts on both transcription and translation in bacterial cells. At the same time, the role of post-translational control in adaptation to nutrient-poor environments is poorly understood. Previous studies demonstrate the ability of the glucosyltransferase UgtP to influence cell size in response to nutrient availability. Under nutrient-rich medium, interactions with its substrate UDP-glucose promote interactions between UgtP and the tubulin-like cell division protein FtsZ in *Bacillus subtilis*, inhibiting maturation of the cytokinetic ring and increasing cell size. In nutrient-poor medium, reductions in UDP-glucose availability favor UgtP oligomerization, sequestering it from FtsZ and allowing division to occur at a smaller cell mass.

**Results:**

Intriguingly, in nutrient-poor conditions UgtP levels are reduced ~ 3-fold independent of UDP-glucose. *B. subtilis* cells cultured under different nutrient conditions indicate that UgtP accumulation is controlled through a nutrient-dependent post-translational mechanism dependent on the Clp proteases. Notably, all three *B. subtilis* Clp chaperones appeared able to target UgtP for degradation during growth in nutrient-poor conditions.

**Conclusions:**

Together these findings highlight conditional proteolysis as a mechanism for bacterial adaptation to a rapidly changing nutritional landscape.

**Electronic supplementary material:**

The online version of this article (10.1186/s12866-018-1155-2) contains supplementary material, which is available to authorized users.

## Background

As single-celled organisms, bacteria constantly alter their physiology to adapt to their environment. Nutrients in particular can dramatically impact bacterial growth and morphology. *Escherichia coli*, *Salmonella*, and *Bacillus subtilis* cells grow several times faster and are up to three times larger when cultured in nutrient-rich medium than when cultured in nutrient-poor medium [1–3]. Nutrient-dependent increases in cell size appear to be a means of accommodating the concomitant increase in macromolecular biosynthesis at faster growth rates, particularly the additional DNA generated by multifork replication [4, 5].

The nutrient-dependent regulation of biosynthesis has been an area of intense interest for many years. Numerous studies have explored how changes in nutrient composition and growth rate impact transcription and translation, which in large part is a response mediated via accumulation of the signaling molecule guanosine pentaphosphate ((p)ppGpp) [6–9]. Although post-translational regulation has been implicated in adaptation to changes in growth phase (e.g. carbon starvation [10, 11]), how fluctuations in nutritional content and growth rate impact post-translational regulation at the molecular level is poorly defined.

In previous work, we identified a class of division antagonists responsible for coordinating cell size with nutrient availability in *B. subtilis* and *E. coli* [4, 5]. Both organisms employ unrelated, yet functionally similar, glucosyltransferases—UgtP in *B. subtilis* and OpgH in *E. coli*—to inhibit division and increase size during growth in carbon-rich medium [12, 13]. In both cases, binding to their substrate, UDP-glucose, stimulates interaction between UgtP and OpgH and the tubulin-like cell division protein FtsZ. The net result of these interactions is a delay the maturation of the cytokinetic ring and an increase cell size. Loss-of-function mutations in *ugtP* or *opgH* and in genes required for UDP-glucose biosynthesis reduce cell size by as much as 35% during growth in nutrient-rich conditions.

UgtP and OpgH both have additional roles as glucosyltransferases that contribute to cell envelope biogenesis. UgtP transfers glucose from UDP-glucose to diacylglycerol to form the diglucosyl-diacylglycerol membrane anchor for lipoteichoic acid (LTA) [14]. OpgH transfers glucose from UDP-glucose to the periplasm as an initial step toward the synthesis of osmoregulated periplasmic glucans (OPGs) [15]. LTA and OPGs are proposed to have similar functions [16] based on the conservation of enzymes involved in their synthesis, their location within the cellular envelope [17, 18], and their contribution to osmoprotection [13, 19].

In *B. subtilis*, UDP-glucose increases UgtP’s affinity for FtsZ [20]. During growth in nutrient-rich conditions UgtP is localized throughout the cytoplasm, where the largest pool of FtsZ is located, and can also be found at the cytokinetic ring and at cell poles [4]. During growth in carbon-poor conditions or when synthesis of UDP-glucose is disrupted, UgtP self-assembles into large oligomers, sequestering it from FtsZ and permitting division to occur at a reduced cell size [20]. In vitro studies suggest UDP-glucose acts as a molecular rheostat, precisely modulating UgtP’s affinity for itself and FtsZ to coordinate size with growth rate and nutrient availability [20]. Curiously, while the UgtP homolog in the Gram-positive pathogen *Staphylococcus aureus* interacts with FtsZ and other divisome proteins, it does not exhibit the same dynamic localization pattern it does in *B. subtilis* nor does it appear to make a significant contribution to cell size [17].

In addition to UDP-glucose-dependent changes in its affinity for FtsZ, UgtP is also subject to nutrient-dependent changes in concentration. UgtP levels are reduced several-fold during growth in nutrient-poor conditions [4]. Defects in the UDP-glucose biosynthesis pathway have no discernable impact on the intracellular concentration of UgtP, suggesting that nutrient-dependent changes in accumulation are independent of the signaling molecule [4].

The striking difference in UgtP levels, together with previous work suggesting protein turnover might be increased in nutrient-poor conditions [10], prompted us to investigate the mechanism underlying this additional layer of UgtP regulation. Here we report that UgtP nutrient-dependent accumulation is governed by a post-translational mechanism involving all three substrate recognition components of the *B. subtilis* Clp protease system. We find that some of the *clp* chaperone genes are upregulated during growth in nutrient-poor medium, suggesting a possible mechanism for increased UgtP degradation under these conditions. These findings suggest an important role for conditional proteolysis in the nutrient-dependent regulation of cellular processes.

## Results

### UgtP accumulation is subject to nutrient-dependent post-translational regulation

In our initial investigation, we observed that the intracellular concentration of a UgtP-6XHis fusion protein was three to four-fold lower when cells were cultured under carbon-poor conditions, but unaffected by the absence of UDP-glucose [4]. Nutrient-dependent changes in concentration contrast strongly with FtsZ, whose levels are essentially constant across a wide range of growth conditions [21]. The net result is a reduction in the UgtP:FtsZ ratio from ~ 1:2 in LB to as low as ~ 1:8 in minimal medium based on previous calculations of absolute FtsZ and UgtP concentrations per cell [4, 22]. To expand on this observation we measured levels of the same UgtP-His fusion across four different nutrient conditions: Lysogeny Broth [LB], S7_50_ + 1% glucose (minimal glucose), S7_50_ + 1% glycerol (minimal glycerol), and S7_50_ + 1% sorbitol (minimal sorbitol). Under each condition, the respective mass doubling time of this strain (*P*_*ugtP*_*-ugtP-his*) was 22′, 39′, 58′, and 78′.

**Fig. 1:**
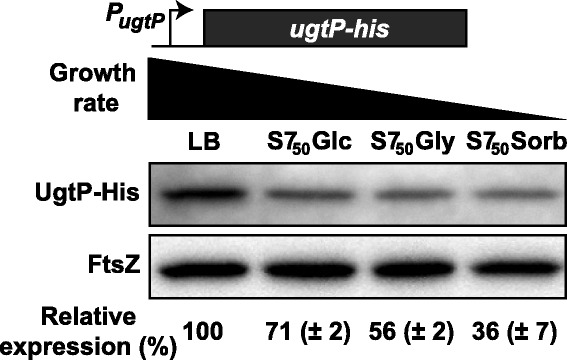
UgtP accumulates only in nutrient-rich conditions. Semi-quantitative immunoblot measuring native UgtP-His levels in a range of growth conditions. PL2265 (*P*_*ugtP*_*-ugtP-his*) was cultured in LB (τ = 22′), minimal glucose (τ = 39′), minimal glycerol (τ = 58′), or minimal sorbitol (τ = 78′). FtsZ is shown as a loading control. Protein levels in LB are set as the reference in the relative expression below (*n* = 3, error = SD)

Consistent with our previous findings, UgtP-His levels increased linearly with nutrient availability and growth rate, as evidenced by a semi-quantitative immunoblot probed with an α-His antibody (Fig. 1). The intracellular concentration of UgtP-His was ~ 3-fold lower in cells cultured in minimal sorbitol, the most carbon-poor condition we examined, than in those cultured in LB, the most nutrient-rich condition examined. To control for the possibility that the His-tag was impacting the stability of UgtP-His, we also measured YFP-UgtP levels in a strain expressing the fusion protein from a xylose-inducible promoter (*P*_*xyl*_*-yfp-ugtP*), cultured in both LB + 0.5% xylose and minimal sorbitol + 0.5% xylose. As we observed with UgtP-His, YFP-UgtP levels were ~ 3-fold lower in minimal sorbitol compared to LB supporting a model in which UgtP (and not the His or YFP tag) is the primary target for degradation during growth in minimal sorbitol medium (Additional file 1:Figure S1).

To determine if UgtP is subject to transcriptional or post-transcriptional modes of regulation, we generated two *ugtP-lacZ* fusion constructs. In the first construct, a reporter for *ugtP* transcription, the 700 base pairs immediately upstream of the *ugtP* start codon were fused to *lacZ*, leaving the *lacZ* Shine-Dalgarno sequence intact. In the second construct, a reporter for UgtP translation, *lacZ* was fused in-frame downstream of the first 30 codons of the *ugtP* open reading frame that included the native *ugtP* Shine-Dalgarno sequence.

**Fig. 2:**
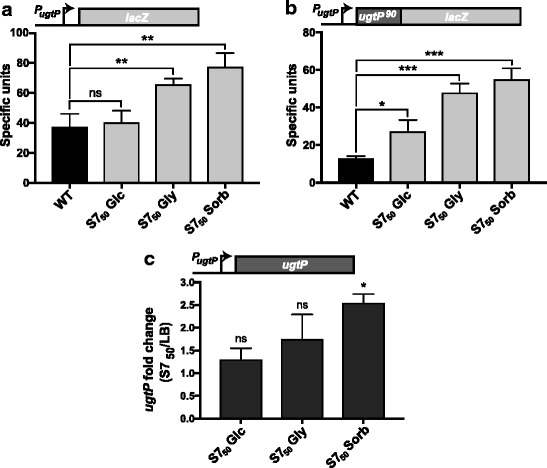
UgtP accumulation is subject to post-translational control. *lacZ* encoding β-galactosidase fused to either **(a)** 700 bp upstream of the *ugtP* start site (*P*_*ugtP*_) to generate a transcriptional fusion (PL1967) or **(b)** an additional 90 bases downstream of the *ugtP* start codon to generate a translational fusion (PL2034). Both strains were cultured in a range of nutrient conditions to generate four different growth rates. Bars indicate mean ± SD of specific β-galactosidase activity (n = 3) and an unpaired T-test was used to access significance (***p < 0.001, ** *p* < 0.01, *p < 0.05, ns *p* > 0.05). **(c)** qRT-PCR measurements of *ugtP* expression levels. Expression in three defined media is normalized to expression in LB. Values are mean ± SD (n = 3). An unpaired T-test was applied to the ∆Ct values to access significance (*p < 0.05, ns p > 0.05)

*lacZ* expression data suggest that nutrient-dependent changes in the intracellular level of UgtP are independent of both transcriptional and translational control. In contrast to UgtP-His levels, expression of both the transcriptional and translational *lacZ* fusions was inversely proportional to growth rate. *lacZ* expression from the transcriptional fusion was 2-fold higher in cells cultured in minimal sorbitol than LB (Fig. 2A) and expression from the translational fusion was 4-fold higher in minimal sorbitol than LB (Fig. 2B). qRT-PCR data also indicated that *ugtP* expression is elevated during growth in minimal sorbitol (Fig. 2C). *ugtP* levels were 1.3-fold higher in minimal glucose, 1.7-fold higher in minimal glycerol, and 2.5-fold higher in minimal sorbitol compared to LB. In all, these data strongly support a model where nutrient-dependent changes in UgtP accumulation are governed by a post-translational mechanism.

### The Clp proteases are responsible for UgtP degradation under carbon-poor conditions

To understand the mechanism responsible for the post-translational regulation of UgtP, we sought to identify the protease responsible for its degradation. As an initial step, we screened *B. subtilis* strains defective in five well-studied proteases: YluC, CptA, ClpP, YvjB, and Lon for aberrant UgtP regulation. We rationalized that if one of these proteases were responsible for the growth rate-dependent degradation of UgtP, then UgtP would inappropriately accumulate in its absence during growth in carbon-poor conditions. For this experiment we used the *P*_*xyl*_*-ugtP-his* construct described above to enhance our ability to detect UgtP by semi-quantitative immunoblot. During growth in LB medium and in the presence of the inducer, xylose, this construct raises intracellular UgtP levels ~ 8.5-fold (changing the UgtP:FtsZ ratio from ~ 1:2 to ~ 4:1) and increases cell length by ~ 22% [4]. (*P*_*xyl*_ is generally insensitive to nutrient availability [21].)

**Fig. 3:**
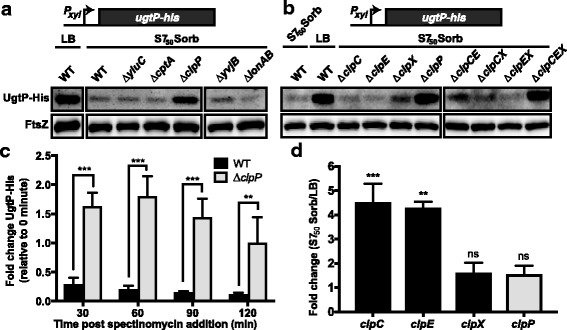
UgtP is subject to nutrient-dependent, post-translational regulation by the Clp proteases. Semi-quantitative immunoblot of UgtP-His expressed from a xylose-inducible promoter (*P*_*xyl*_*-ugtP-his*) in the absence of **(a)** 5 proteases, YluC, CptA, ClpP, YvjB, and Lon (PL2022, PL2028, PL2102, PL2032, and PL2033, BH10 as WT) or **(b)** single/combinatorial deletions of the Clp chaperones (ClpC, ClpE, and/or ClpX) (BH127, BH128, BH130, PL2102, BH135, BH136, BH137, BH138). Cells were cultured in either LB + 0.5% xylose or minimal sorbitol + 0.5% xylose, and immunoblotted against His and FtsZ. Samples shown in **(a)** and also for **(b)** were run on the same blot, but cropped during image processing. **(c)** Fold change in UgtP-His levels for *P*_*xyl*_*-ugtP-his* (BH10) and *P*_*xyl*_*-ugtP-his* + *∆clpP* (BH129) after adding spectinomycin to inhibit translation. Cells were cultured in minimal sorbitol + 0.5% xylose, sampled every 30 min after spectinomycin addition, and subjected to immunoblotting against His antibody. Values are mean ± SD (*n* = 3). A two-way ANOVA was used to assess differences in UgtP-His levels ± *clpP* over time (*P* < 0.0001) and a Bonferroni multiple comparisons test (shown) was used to determine significance between the two strains at specific time intervals (***p < 0.001, **p < 0.01). **(d)** qRT-PCR measurements of *clpC*, *clpE*, *clpX*, and *clpP* expression in minimal sorbitol versus LB. Values are mean ± SD (*n* = 4). An unpaired T-test was applied to the ∆Ct values to access significance (***p < 0.001, **p < 0.01, ns *p* > 0.05)

Analysis of the protease-deficient strains strongly implicated ClpP in the nutrient-dependent control of UgtP accumulation. UgtP-His levels increased significantly while cultured in minimal sorbitol only in the strain defective for ClpP (Fig. 3A). UgtP-His levels were approximately 5-fold higher during growth in minimal sorbitol in the ∆*clpP* strain than in the parental strain or in the four other protease mutants. ClpP-dependent proteolysis distinguishes UgtP from FtsZ, which is insensitive to Clp-mediated proteolysis in *B. subtilis* [23, 24].

ClpP is a processive serine protease widely conserved throughout bacteria. Ineffective on its own, ClpP functions in tandem with AAA+ ATPase Clp chaperones [25]. The Clp chaperones are responsible for recognizing target proteins, unfolding them using the energy of ATP hydrolysis, and translocating the unfolded polypeptide into the ClpP proteolytic chamber [25, 26]. In *B. subtilis*, there are three Clp chaperones: ClpC, ClpE, and ClpX. Since each chaperone identifies a unique set of substrates with limited target overlap, we reasoned that either ClpC, ClpE, or ClpX would be responsible for growth rate-dependent degradation of UgtP. To distinguish the Clp chaperone involved, we examined accumulation of UgtP-His from a xylose-inducible promoter in ∆*clpC*, ∆*clpE*, and ∆*clpX* cells cultured in minimal sorbitol.

Deletion of *clpC*, *clpE*, and *clpX* alone or in pairwise combination had little impact on UgtP-His levels in minimal sorbitol. UgtP-His accumulated to levels similar to that observed in the ∆*clpP* mutant only in a strain defective for all three chaperones (Fig. 3B). These data suggest that all three Clp chaperones function redundantly to control UgtP accumulation in a growth rate-dependent manner.

To confirm that the Clp proteases are indeed responsible for degradation of UgtP, as our data suggested, we employed an in vivo proteolysis assay similar to that described in [27]. Briefly, we cultured *P*_*xyl*_*-ugtP-his* and the congenic *∆clpP* strains in minimal sorbitol with xylose to an A_600_ of ~ 0.2. We then added chloramphenicol to inhibit new protein synthesis and sampled cells every 30 min for 2 h. UgtP-His levels were determined via semi-quantitative immunoblot. Consistent with UgtP being directly targeted by ClpP, UgtP-His levels decreased rapidly in *clpP*^*+*^ cells over time, but were stable in the absence of the protease (Fig. 3C and Additional file 2**:** Figure S2).

Consistent with the Clp proteases serving as nutrient-dependent regulators of protein stability, expression of *clpC* and *clpE* were elevated in minimal sorbitol (Fig. 3D). qRT-PCR indicated that expression of *clpC* and *clpE* are upregulated > 4-fold, while *clpX* and *clpP* are marginally upregulated (~ 1.5-fold) in minimal sorbitol, our poorest carbon source, relative to LB. These findings are consistent with those of the Hecker lab who observed ClpXP and ClpCP drive proteolysis of a large group of proteins, most notably enzymes involved in central carbon metabolism, upon glucose starvation (UgtP was not identified as a Clp substrate in this study) [10].

### Defects in UgtP’s hexose-binding site increases susceptibility to proteolysis in vivo

In an attempt to identify regions of UgtP required for recognition by the Clp chaperones, we screened a series of UgtP mutants for sensitivity to Clp-mediated degradation in vivo. Of particular interest were regions of UgtP that mediate interaction with UDP-glucose, FtsZ, or itself. To test the impact of UDP-glucose binding and oligomerization on Clp-dependent degradation, we employed three UgtP mutants, generated based on structural data from the UgtP homologue, mongalactosyldiacylglycerol (MGDG) synthase [28]: one defective in its putative uracil-binding site (URA-: F112A V117A), one defective in the putative hexose-binding site (HEX-: E306A N309A), and one in the predicted dimerization/oligomerization domain (OLI-: I142A E146A).

**Fig. 4:**
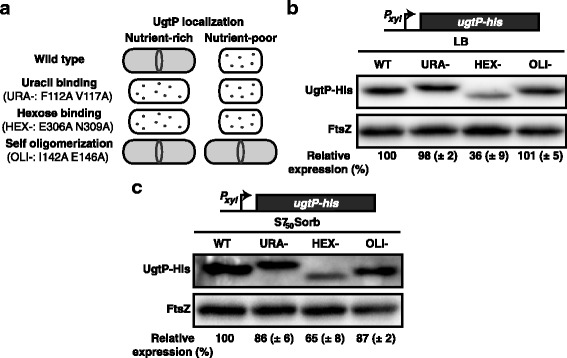
Mutations in UgtP’s putative hexose-binding site enhance susceptibility to proteolysis in vivo. **(a)** A schematic representation of YFP-UgtP localization and cell size for wild type, uracil-binding (URA-), hexose-binding (HEX-), and self-oligomerization (OLI-) mutants in either LB or minimal sorbitol (Additional file 3:Figure S3 & [20]). UgtP-His variants BH736 (WT), BH742 (URA-), BH752 (HEX-), BH740 (OLI-) were cultured in either **(b)** LB + 0.5% xylose or **(c)** minimal sorbitol + 0.5% xylose and subjected to semi-quantitative immunoblotting against His and FtsZ (loading control) antibodies. Relative expression compared to WT (BH736) UgtP-His is shown (*n* = 3, error = SD)

All three UgtP mutants behaved as predicted based on structural and biochemical data from the MGDG synthase (Fig. 4A) [28]. Both UgtP^URA-^ and UgtP^HEX-^ are defective as sugar transferases, exhibit a punctate localization pattern during growth in nutrient-rich medium, and fail to complement a *ugtP* null strain for cell size, all of which is consistent with increased self-association and a loss of interaction with FtsZ [20]. In support of our model that oligomerization serves to sequester UgtP from FtsZ, during growth in the absence of UDP-glucose the putative oligomerization mutant (UgtP^OLI-^) exhibited less punctate (self-associated) localization (Additional file 3: Figure S3A) and antagonized division to increase cell length by more than 30% (Additional file 3: Figure S3B).

For analysis of UgtP mutant accumulation, His-tagged versions of all three UgtP variants (URA-, HEX- and OLI-) were expressed from an ectopic locus under the control of a xylose-inducible promoter in a *ugtP* null background. UgtP mutant accumulation under nutrient-rich (LB) and carbon-poor conditions (minimal sorbitol) was monitored by semi-quantitative immunoblot in early exponential phase. For reasons that are unclear, both UgtP^URA-^-His and UgtP^HEX-^-His exhibited migration patterns different from wild type UgtP-His when separated by SDS-PAGE prior to immunoblotting (Fig. 4). This difference may reflect conformational changes in mutant protein structure.

Of the three mutants, only UgtP^HEX-^-His exhibited ClpP-dependent differential in accumulation compared to wild type UgtP-His, suggesting hexose binding might protect UgtP from proteolysis. UgtP^HEX-^-His accumulated to only ~ 35% of UgtP-His (WT) levels during growth in LB (Fig. 4B). Even in minimal sorbitol, UgtP^HEX-^-His levels were ~ 65% of UgtP-His (Fig. 4C). UgtP^URA-^-His and UgtP^OLI-^-His were indistinguishable from wild type UgtP-His with regard to ClpP-dependent changes in accumulation, arguing against a role for interaction with FtsZ or homo-oligomerization in shielding UgtP from Clp recognition. Importantly, each of the constructs exhibited congruent mRNA levels measured by qRT-PCR and UgtP^HEX-^-His is at WT levels in a ∆*clpP* background strain in both LB and minimal sorbitol, indicating that the change in stability is directly due to ClpP-mediated degradation (Additional file 4: Figure S4).

Together these data suggest that interaction with the hexose moiety of UDP-glucose, which we have measured as being more prevalent when cultured in nutrient-rich conditions (Additional file 5: Figure S5), may provide some protection from Clp-mediated proteolysis. At the same time, however, this observation is inconsistent with our previous observation that UgtP accumulation is independent of UDP-glucose synthesis [4], a point we address in the discussion.

### UgtP is targeted by ClpXP for proteolysis in vitro

To clarify whether Clp recognition of UgtP is direct or reliant on a nutrient-dependent adaptor protein, we next determined if UgtP could be degraded directly by ClpXP in vitro. Although our genetic data indicate all three Clp chaperones are capable of degrading UgtP (Fig. 3B), we focused on ClpX as the most prevalent chaperone (there are an estimated 1400 ClpX, 250 ClpC, and 100 ClpE hexamers per cell during growth in LB) [29].

For these experiments, we expressed and purified ClpX and ClpP as previously described and a His-UgtP we constructed for this study [24]. We employed the well-characterized ClpXP substrate Spx [30] as a positive control, and a non-targeted protein, Thioredoxin-His, as a negative control. As expected, Spx was subject to robust ATP-dependent ClpXP mediated degradation, while Thioredoxin-His was retractile to ClpXP proteolysis (Additional file 6: Figure S6). Due to the size-similarity between His-UgtP and ClpX used in the degradation assays (44 kDa vs 46 kDa), we could not distinguish between the two when separated by standard SDS-PAGE. Instead, we employed a monoclonal anti-His antibody to distinguish between His-UgtP and ClpX via immunoblot. Please note that because we detected UgtP by immunoblot and the anti-His antibody (monoclonal THE™ antibody, Genscript) interacted strongly with nanogram amounts of the His-UgtP fusion protein, we found it necessary to utilize significantly lower concentrations of His-UgtP relative to the Spx and Thioredoxin-His control proteins to remain in linear range. While His-UgtP levels were monitored by immunoblot, Spx and Thioredoxin-His were monitored by Coomassie staining.

**Fig. 5:**
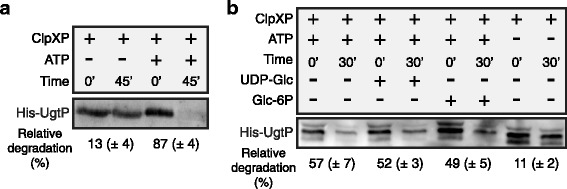
ClpXP targets UgtP for proteolysis in vitro independent of UDP-glucose. **(a)** Immunoblot of purified His-UgtP after incubation with purified ClpXP. Reactions consisting of 3 μM ClpX, 6 μM ClpP, 3 μM His-UgtP, and 5 mM ATP were incubated for 45 min at room temperature. ClpXP substrate controls are shown in Additional file 6: Figure S6. **(b)** In vitro ClpXP cleavage assay ± UDP-glucose or glucose-6P. The assay used 3 μM ClpX, 6 μM ClpP, 3 μM His-UgtP, 5 mM ATP, and 2 mM of either UDP-glucose or glucose-6P. α-His was used to visualize His-UgtP levels by immunoblot. Each cognate set was used to gauge relative degradation (%) as shown below (n = 3, error = SD)

Consistent with our genetic analysis, in vitro data strongly support a model in which UgtP is degraded by ClpXP facilitated through a direct interaction between this Clp complex and UgtP (Fig. 5A). Incubating purified His-UgtP with the ClpXP complex without ATP resulted in minimal degradation (13%). In stark contrast, when ATP was added to reactions 88% of His-UgtP was proteolyzed. Though the possibility of contaminating non-specific ATP-dependent proteases in the purified protein preparations contributing to the observed His-UgtP degradation exists, we did not observe any degradation of our negative control, Thioredoxin-His (Additional file 6: Figure S6).

Based on our in vivo data supporting a model in which hexose binding may shield UgtP from Clp-mediated proteolysis (Fig. 4), we speculated that adding UDP-glucose or simply glucose to the ClpXP proteolysis assays might hinder UgtP degradation. To test this model we added UDP-glucose or glucose 6-phosphate in excess to the ClpXP in vitro proteolysis reactions described above. However, we saw no difference in UgtP proteolysis in the presence of either sugar (Fig. 5B).

### Clp-mediated UgtP degradation during growth in minimal sorbitol medium does not significantly impact cell size or diglucosyl-diacylglycerol production

In an effort to illuminate the potential “rationale” for Clp mediated degradation of UgtP during growth in carbon-poor medium we examined the impact of Clp activity on cell size and production of diglucosyl-diacylglycerol (DGD), the anchor for LTA. UgtP exhibits a high affinity for FtsZ (38 nM) even in the absence of UDP-glucose [20]. Nutrient-dependent degradation may thus serve as an additional control to prevent UgtP-mediated division inhibition during growth in carbon-poor conditions.

For these experiments, we took advantage of two strains capable of producing a range of UgtP concentrations in minimal sorbitol: BH736 (*∆ugtP; P*_*xyl*_*-ugtP-his*) and BH12 (*P*_*ugtP*_*-ugtP-his; P*_*xyl*_*-ugtP-his*). Semi-quantitative immunoblots indicate that non-induced BH736 produces no detectible UgtP-His, induced BH736 and non-induced BH12 produce similar levels of UgtP-His, and induced BH12 produces approximately 60% more UgtP-His than BH12 non-induced (Additional file 7: Figure S7). If cell size under carbon-poor conditions is sensitive to UgtP concentration, then we would expect to see a gradient of cell sizes with cell size increasing in proportion to UgtP concentration. We opted not to measure the impact of ClpP on cell size since *clpP* is highly pleotropic and deletion could feasibly alter cell length independent of UgtP degradation.

**Fig. 6:**
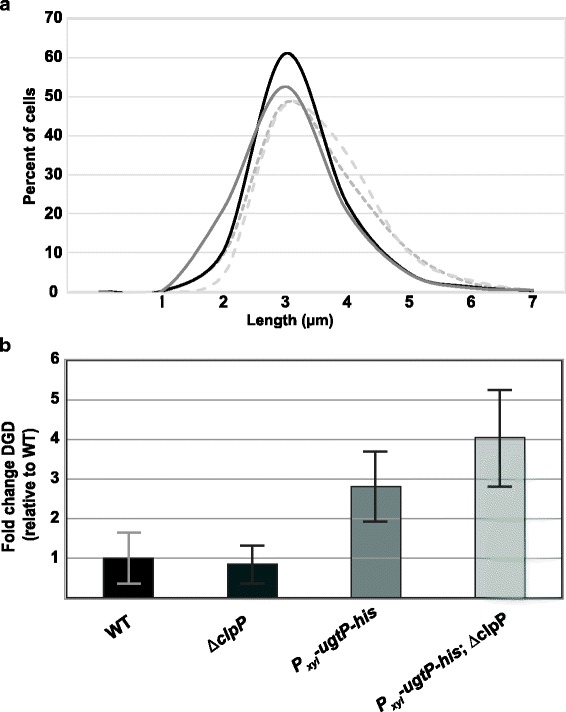
UgtP levels do not significantly affect cell length or diglucosyl-diacylglycerol levels in carbon-poor media. **(a)** Cell length distributions of strains BH736 (*∆ugtP; P*_*xyl*_*-ugtP-his*) and BH12 (*P*_*ugtP*_*-ugtP-his; P*_*xyl*_*-ugtP-his*) cultured in minimal sorbitol ±0.5% xylose, determined by measuring the distance between the mid-points of adjacent cell wall septa (*n* = 600, error = SD). **(b)** Relative diglucosyl-diacylglycerol concentrations of lipid extracts from strains PL522 (WT), PL2102 (*∆clpP*), BH10 (*P*_*xyl*_*-ugtP-his*), BH129 (*P*_*xyl*_*-ugtP-his; ∆clpP*) cultured in minimal sorbitol, determined by thin layer chromatography and subsequent densitometric analysis of separated lipids (n = 3, error = SD)

Somewhat surprisingly, we did not observe a significant difference in cell size between strains regardless of the presence of xylose. Average cell size was not significantly different between any set of conditions, including the UgtP-null (BH736 –xylose) and UgtP-overexpression conditions (BH12 + xylose) (Table 1, *p* = 0.28). Additionally, cell size distributions for each condition are not obviously different from one another (Fig. 6A). These data fail to support a model in which Clp-mediated UgtP degradation is important for maintaining cell size in carbon-poor medium.

**Table 1:** Cell length in response to differential UgtP levels in minimal sorbitol

Strain	Relative UgtP Expression	Average Cell Length [μm]	Change in Length (%)
BH736 – xylose (*∆ugtP; P*_*xyl*_*-ugtP-his*)	0	2.63 ± 0.27	Reference
BH736 + xylose (*∆ugtP; P*_*xyl*_*-ugtP-his*)	99 ± 45	2.72 ± 0.11	+ 3.4%
(*P*_*ugtP*_*-ugtP-his; P*_*xyl*_*-ugtP-his*) BH12 – xylose	100 ± 24	3.07 ± 0.20	+ 16.7%
(*P*_*ugtP*_*-ugtP-his; P*_*xyl*_*-ugtP-his*) BH12 + xylose	155 ± 33	2.95 ± 0.25	+ 12.2%

Average cell length of strains BH736 (*∆ugtP; P*_*xyl*_*-ugtP-his*) and BH12 (*P*_*ugtP*_*-ugtP-his; P*_*xyl*_*-ugtP-his*) cultured in minimal sorbitol ±0.5% xylose, determined by measuring the distance between the mid-points of adjacent cell wall septa (n = 600, error = SD). Relative UgtP expression is demonstrated in Additional file 7: Figure S7

Another possible explanation for the degradation of UgtP in carbon-poor conditions is the preservation of intracellular glucose by curtailing production of UgtP’s product, diglucosyldiacylglycerol [14]. To test this possibility we measured DGD levels in wild type and ∆*clpP* strains, as well as their isogenic *P*_*xyl*_*-ugtP* counterparts, cultured in minimal sorbitol (with xylose added when growing *P*_*xyl*_*-ugtP* strains). Briefly, we purified membranes from these strains, performed a methanol:chloroform lipid extraction, separated the lipids using thin-layer chromatography, stained for DGD using iodine gas, and quantified DGD. Lipids were also extracted from *∆ugtP* cells for use as a negative control.

Despite the 3-fold difference in UgtP concentration, DGD levels were not significantly different between WT/*∆clpP* cells (*p* = 0.78) or induced *P*_*xyl*_*-ugtP* cells ± *clpP* (*p* = 0.30) (Fig. 6B). As expected, *∆ugtP* cells produced no detectible DGD (Additional file 8: Figure S8). These data suggest that while UgtP is necessary for DGD production, degradation of UgtP via the Clp proteases is irrelevant for modulating DGD concentration. It is therefore unlikely that degradation of UgtP occurs in carbon-poor conditions as a means of conserving glucose, as production of glucose-rich DGD is not affected by UgtP degradation.

## Discussion

Our results indicate UgtP accumulation is controlled in a nutrient-dependent manner via a post-translational, Clp-dependent mechanism. This additional layer of regulation governing UgtP activity, distinct from UDP-glucose-mediated changes in UgtP’s affinity for itself and FtsZ, ensures that active UgtP accumulates only under nutrient-rich growth conditions when it antagonizes cell division. While there are several prominent examples of conditional proteolysis coupled to growth phase [31–33], there are only a handful of examples in which proteolysis has been linked to growth rate and/or nutrient availability. Two such examples are from the Narberhaus lab: 1) The DNA-binding replication inhibitor CspD is selectively proteolyzed by the Lon protease during growth in nutrient-rich medium [34] and 2) LpxC, a deacetylase involved in lipid A biosynthesis, is degraded only when cultured at slower growth rates by the protease FtsH [35].

We were somewhat surprised by our finding that UgtP proteolysis in vivo is accomplished via the redundant activity of ClpCP, ClpEP, and ClpXP (Fig. 3B). While ZapC is recognized and degraded by ClpXP and ClpAP in *E. coli*, to our knowledge UgtP is the first published example of a Clp substrate recognized by all the Clp chaperone proteins in *B. subtilis* [36]. Further, we find that genes encoding two of the *clp* chaperones, *clpC* and *clpE*, are expressed at higher levels during slower growth, which likely contributes toward the growth medium-dependent regulation of UgtP (Fig. 3D). It is noteworthy that ClpX, previously identified as a direct inhibitor of FtsZ assembly in *B. subtilis* independent of its role in proteolysis, also modulates the stability of an entirely different FtsZ inhibitor [24].

Since AAA+ proteases exhibit distinct substrate-binding repertoires with only minor overlap in target specificity [37], it is curious how and why all three Clp chaperones are able to target UgtP. These chaperones either recognize unstructured peptide sequence tags (known as degrons) within a client protein or adaptor proteins bound to target substrates. ClpC exclusively uses adaptor proteins (MecA, YpbH, and McsB) to recognize substrate as well as to promote the activation of the ClpC hexamer [38–41]. Although ClpXP is capable of degrading UgtP in vitro (Fig. 5), we were unable to identify putative degrons for any of the three Clp chaperone proteins within the UgtP primary sequence through comparison with other verified and putative *B. subtilis* Clp substrates [10, 42, 43]. It thus remains an open question whether ClpXP, ClpCP, and ClpEP share a common degron or adaptor protein, or use distinct mechanisms, to target UgtP for degradation.

UgtP degradation was enhanced in vivo by two point mutations (E306A N309A) in its putative hexose-binding site suggesting ligand binding might afford some protection from ClpP-specific proteolysis (Fig. 4). While there is precedent in both prokaryotic and eukaryotic systems of small molecule ligands governing proteolytic susceptibility [44–46], our data do not cohesively support that UDP-glucose shields UgtP from degradation. Neither defects in UDP-glucose biosynthesis [4] nor mutations disrupting the putative nucleotide binding site (URA-, F112A V117A) had an impact on UgtP accumulation (Fig. 4). Instead, these data suggest ligand binding is largely irrelevant to UgtP stability and the hexose-binding mutation (E306A N309A) may simply lead to a conformation that exposes a Clp recognition sequence. Although we are unaware of any literature supporting a negative relationship between *clp* expression or activity and cell size, enrichment of the Clp proteases—particularly ClpC and ClpE—under nutrient-poor conditions as the most parsimonious, albeit still theoretical, explanation for UgtP degradation under these conditions (Fig. 3D).

## Conclusions

As a whole, our data point to UgtP being subject to an elaborate set of multilayered controls. Not only is it unclear why three Clp chaperones would be needed for UgtP degradation in minimal medium, it is also not readily apparent why a carbon-starved cell would spend energy to transcribe and translate *ugtP* only to immediately have it proteolyzed, consuming ATP at each step. We were unable to identify a phenotypic explanation for the nutrient-dependent degradation of UgtP. Given the myriad of conditions under which *B. subtilis* is able to grow, it may very well be that we are looking in the wrong place and for the wrong phenotypes. Alternatively, UgtP may simply be one of a large cohort of proteins that have a shorter half-life during growth in carbon-poor conditions [47]. If so, growth medium-dependent changes in Clp protease activity may simply serve as a crude means of supporting *B. subtilis’* uncanny ability to adapt rapidly to a wide range of environments while ensuring cells retain a relatively robust supply of biosynthetic building blocks even during growth in carbon-poor conditions.

## Methods

### Strains and general methods

All *B. subtilis* strains are derivatives of JH642 [48]. Details of their construction and a list of strains used for this study are described in (Additional file 9 and Additional file 10: Table S1). All cloning was done using the *E. coli* strain AG1111 [49]. Either Vent (NEB) or Phusion (NEB) DNA polymerases were used for PCRs. Cells were cultured in either Lysogeny Broth (LB) or minimal S7_50_ defined media [50] supplemented with either 1% glucose, glycerol, or sorbitol as carbon sources and appropriate amino acid supplements. For strains containing *∆clpP* or *∆clpCEX*, cells were always first cultured in LB prior to growth in other growth media.

### Semi-quantitative immunoblotting

Bacterial cells cultured to A_600_ 0.20–0.40 were lysed by centrifuging 1 mL of culture then re-suspending in 50 μL lysis buffer (20 mM tris pH 8.0, 12.5 mM MgCl_2_, 1 mM CaCl_2_, 2 mg/mL lysozyme, 1X Halt™ Protease Inhibitor Cocktail), incubating 10 min at 42 °C, then adding SDS to 1% (*v*/v). Laemmli buffer was added to 1X, lysates were incubated 10 min at 100 °C, then lysate was normalized to OD and subjected to SDS-PAGE. Proteins were transferred to PVDF membranes using Bio-Rad Trans-Blot® Turbo™ instrument as described on pg. 15 of the operation manual. Subsequent to transfer, but prior to blocking PVDF membranes were stained with 1X Ponceau S solution in 5% acetic acid for 5 min and total protein per lane used as a loading control [51]. Membranes were then blocked with 5% nonfat milk in PBS for 1 h.

Two different Anti-His antibodies to detect His-tagged UgtP were employed in this study: 1) His-probe monoclonal antibody (Santa Cruz Biotechnology) used at 1:1000 (used in Fig. 3), and 2) monoclonal THE™ His Tag (Genscript) α-His antibodies incubated at 1:1000. Rabbit polyclonal (Genscript) α-GFP antibodies incubated (at 1:1000) were used to detect YFP-tagged UgtP. FtsZ was detected using an affinity-purified polyclonal rabbit α-FtsZ antibody (at 1:5000) [52]. Cognate goat α-rabbit or goat α-mouse (Genscript) HRP secondary antibodies were used (at 1:5000). The membranes were developed using ECL substrate (Bio-Rad #1705060) and imaged using a Li-COR Odyssey FC instrument that detected saturation and autocorrected exposure time. Loading was first normalized to cell density measured by A_600_, then total protein per lane was calculated using Ponceau-S prior to blocking. Blot density was quantified using ImageJ (NIH) by subtracting background and normalizing to total protein (Ponceau S staining). In some cases, FtsZ is shown as the loading control because it was largely invariant with Ponceau-S staining.

### In vivo UgtP degradation assay

Protocol was adapted from [27]. Strains were cultured in 5 mL LB + 0.5% xylose at 37 °C with shaking until A_600_ = 0.20–0.40. These cultures were then diluted into 20 mL minimal sorbitol + 0.5% xylose to A_600_ = 0.005. Once A_600_ reached 0.20–0.40, spectinomycin was added to 200 μg/mL. Cultures were sampled (1 mL) at 0, 30, 60, 90, and 120 min after spectinomycin addition, and the samples were frozen at − 80 °C for future use. These samples were then subjected to semi-quantitative immunoblotting for UgtP-His, the protein was quantified using ImageJ (NIH) and processed in Microsoft Excel.

### β-galactosidase activity in liquid cultures

Specific activity was calculated essentially as described in [53]. Strains encoding *lacZ* fusions were cultured at 37 °C to early/mid-log (A_600_ 0.30–0.40). Prior to sampling, the cultures were diluted 1:2 in their respective medium and absorbance at A_600_ was recorded. 30 μl of toluene and 30 μl of a 0.1% sodium dodecyl sulfate solution were added to 2 ml of bacterial culture to permeabilize cells. Incubating cells at 37 °C for 45 min then evaporated the toluene. Cells were then mixed with Z-buffer (60 mM Na_2_HPO_4_, 40 mM NaH_2_PO_4_, 10 mM KCl, 1 mM MgSO_4_, 50 mM β-mercaptoethanol) and tubes were incubated at 25 °C for 5′. Reactions were started by adding 0.25 ml of 0.4% o-nitrophenol-β-galactoside in Z-buffer and stopped by adding 0.5 ml of 1 M Na_2_CO_3_. A_420_ was then recorded. The specific unit value was calculated using the equation: = 200 × (A_420_ of the culture − A_420_ in the control tube)/minutes of incubation × dilution factor.

### Quantitative real-time polymerase chain reaction

RNA was harvested from *B. subtilis* cells in early-log (A_600_ = 0.20–0.40) with RiboPure™ Kit (Ambion), treated with the Turbo DNA-Free Kit™ (Ambion), and reverse transcribed for 1 h at 42 °C using the RETROscript® Kit (Ambion). Template was diluted 10-fold, added to iTaq SYBR Green Supermix (Bio-Rad) with appropriate oligonucleotide pairs. Oligonucleotides used in this study are described in (Additional file 11: Table S2). Data were acquired using an Applied Biosystems model 7500 thermocycler. Results were analyzed using the comparative Pfaffl method [54].

### Protein purification

All plasmids encoding genes used for purification were mini-prepped from storage *E. coli* strain AG1111 and freshly transformed into C41(DE3) cells and consequently used for expression of protein (no frozen stocks were used). Briefly, 1 L of LB medium was inoculated 1:100 with overnight culture from a single colony. Cells were grown at 37 °C with the exception of ClpP and ClpX, which were grown at room temperature. When A_600_ ~ 0.6, cells were induced with isopropyl 1-thio-β-D-galactopyranoside to a final concentration of 1 mM. Cells were grown for an additional 4-8 h and then cells were harvested by centrifugation, and cell pellets were stored at − 80 °C.

ClpP and ClpX were purified using the IMPACT System (NEB) as described previously [24]. Spx was also purified as previously described [24] with the following modifications: Spx-His was purified from a Ni-NTA column with a 50-500 mM imidazole gradient and peak fractions pooled. An N-terminal 6X–His tag was then removed by cleavage with AcTev protease (Life Technologies). Spx was further purified using size exclusion chromatography over an S-300 column in buffer containing 50 mM Tris pH 7.5, 50 mM KCl, and 10% glycerol. Peak fractions were collected, pooled, and concentrated using an Amicon-10 kDa centrifugation column, flash frozen on liquid nitrogen, and stored at − 80 °C.

His-UgtP in pET28a(+) (PL3521) was purified as follows: Starting from frozen pellets, cells were thawed on ice and re-suspended in Buffer A (50 mM Tris pH 8.0, 500 mM NaCl, 10 mM Imidazole, 10% glycerol). Pefabloc-SC (Sigma) was then added as a protease inhibitor. Re-suspended cells were then lysed by three times by French press at a pressure of 10,000 psi. The lysed cells were then cleared by centrifugation, spinning at 120,000×g for 45 min at 4 °C. The resulting supernatant was brought up to a volume of 50 mL and loaded onto a DynaLoop connected to a DuoFlow F10 FPLC system (Bio-Rad). The supernatant was then applied over two 5 mL Bio-Scale Mini Profinity IMAC cartridges connected in series (Bio-Rad). The columns were then washed with 10 column volumes of Buffer A followed by 5 column volumes of Buffer B (50 mM Tris pH 8.0, 500 mM NaCl, 20 mM Imidazole, 10% glycerol). Protein was then eluted off of the columns with 5 column volumes of Buffer C (50 mM Tris pH 8.0, 500 mM NaCl, 500 mM Imidazole, 10% glycerol). Peak fractions were collected and concentrated to a volume of ~ 1 mL using an Amicon-10KDa centrifugation column. The concentrated protein was loaded onto a 1 mL loop and applied over a HiPrep 16/60 Sephacryl S-300 HR size exclusion column (GE Healthcare). The column was washed and protein was then eluted off the column in Buffer D (50 mM HEPES pH 7.5, 100 mM KCl, 10% glycerol). Peak fractions were checked by SDS-PAGE and then aliquot, flash frozen on liquid nitrogen and stored at − 80 °C.

### In vitro ClpXP proteolysis assay

Reactions were carried out in 50 mM HEPES pH 7.6, 50 mM KCl, 15 mM Mg acetate, 5 mM DTT, 5 mM ATP, 10 mM creatine phosphate, and 0.1 μg/μl creatine kinase (Sigma). 500 ng of His-UgtP was incubated at 37 °C in the presence of ClpP (6 μM) and ClpX (3 μM) in a 100 μl reaction mixture. Each reaction was initiated by the addition of 5 mM ATP. At 0 and 30 or 45 min, samples of the reactions were taken containing what corresponded to 125 ng His-UgtP at the start of the reaction. Samples were then diluted to 2.5 ng/μl with 2X sample buffer. 50 ng of His-UgtP was then loaded per lane on a 10% SDS-PAGE. After electrophoresis, proteins were transferred via Western blot to a PVDF membrane. Membranes were blocked, then incubated with mouse monoclonal THE™ His Tag (Genscript) at a 1:4000 dilution overnight at 4 °C. Control reactions with Spx and Thio-His were performed as with His-UgtP except at a concentration of 3 μM. At 0 and 60 min, 15 μl of the samples was collected and analyzed on a 15% SDS-PAGE followed by staining with Coomassie blue.

### Fluorescence microscopy and cell length measurements

Samples were first fixed as previously described [55]. Briefly, cells were fixed by treating with 2.575% paraformaldehyde and 0.0008% glutaraldehyde. Fixed samples were permeabilized with 0.01 mg/mL lysozyme for two minutes, and then treated with 10 μg/mL wheat germ agglutinin tetramethylrhodamine conjugate (WGA-Rhod, ThermoFisher #W849) in PBS to stain cell wall septa for 10 min. 5 μL of prepared samples were applied to 1% agarose in PBS pads, allowed to dry, and then covered with a coverslip. Cells were imaged using a Nikon TI-E microscope equipped with a Texas Red/mCherry/AlexaFluor 594 filter set for fluorescence microscopy (Chroma # 39010).

Cell length was determined using Nikon Elements by measuring the distance between the midpoints of adjacent cell wall septa for cells stained with WGA-Rhod. T-test analysis was employed to test for significant differences between conditions.

### Lipid extraction

Bacterial cultures (1 L volumes) were centrifuged at 15,000 x g for 1 min, decanted, and then resuspended in 100 mL 100 mM sodium citrate pH 4.7. Cells were lysed using a French press. Lysates were centrifuged at 12,000 g for 20 min to pellet membranes. Pellets were weighed and resuspended to 0.4 g/mL in 100 mM sodium citrate pH 4.7. Methanol and chloroform were added to obtain a ratio of 2:1:0.8 methanol:chloroform:sodium citrate. Mixture was vortexed 30 s every 15 min for 2 h. Chloroform and sodium citrate were added to obtain a ratio of 1:1:0.9 methanol:chloroform:sodium citrate. The mixture was vortexed for 1 min and centrifuged at 4000 x g for 10 min. Bottom chloroform layer was transferred to a new tube. Methanol and sodium citrate were added to obtain a ratio of 1:1:0.9 methanol:chloroform:sodium citrate. Mixture was vortexed for 1 min and centrifuged at 4000 x g for 10 min. Bottom chloroform layer was transferred to new tube and allowed to dry in fume hood. Dried lipids were weighed and resuspended in 1:1 methanol:chloroform to a final concentration of 600 mg/mL and stored at − 20 °C.

### Lipid thin-layer chromatography and diglucosyl-diacylglycerol quantification

140 mL chloroform and 60 mL methanol were added to a glass TLC development chamber. Placed filter paper into developing solution, thoroughly wetted blot paper, and then placed the paper along the chamber walls. A TLC plate (Analtech #p46021) was pre-run in developing solution by placing it in the chamber, covering the chamber with its glass lid, and allowing the solvent to run to the top of the plate. The plate was dried at 100 °C for 10 min. Using a pencil, a line was drawn across the width of the plate approximately 1 cm from the bottom. Lipid samples (2 μL) were spotted along this line and allowed to dry. Samples were repeatedly spotted and allowed to dry 5 additional times. Once the spots were dry, the plate was placed back into the chamber, the lid was placed on top, and the solvent was allowed to run to 1 cm from the top of the plate. The plate was taken out of the development chamber, the solvent line was quickly marked with pencil, and the plate was dried. The plate was then placed inside a polystyrene container with a few iodine crystals, sealed, and stained for 16 h with iodine gas. The stained plate was scanned to acquire images. The stained plate was scanned to acquire images. ImageJ was used to quantify the amount of DGD present, and another band (indicated in Additional file 8: Figure S8) was used as a loading control. T-test analysis was employed to test for significant differences between conditions.

### Statistical analysis

Statistical parameters and significance are reported in the figures and the figure legends. Asterisks designate statistical significance as: *, *p* < 0.05; **, *p* < 0.01; ***, *p* < 0.001; ****, *p* < 0.0001. Statistical analysis was performed in GraphPad PRISM 6.

## Additional files

Additional file 1: **Figure S1.** YFP-UgtP is degraded in minimal sorbitol; this file shows ectopically expressed YFP-UgtP cultured in nutrient-rich and nutrient-poor media. (PDF 817 kb)

Additional file 2: **Figure S2.** Representative immunoblot for in vivo UgtP degradation experiment; this file shows an immunoblot of UgtP-His from cells with and without *clpP*, cultured in nutrient-poor media over a 2-h period, after addition of spectinomycin. (PDF 967 kb)

Additional file 3: **Figure S3.** UgtP oligomer mutant has WT localization and delays cell division in a Δ*pgcA* background; this file shows immunofluorescence images of cells harboring either *yfp-ugtP* or *yfp-ugtP*^*OLI-*^ in a UDP-glucose null background cultured in nutrient-rich media, and also shows cell length distributions for the previously mentioned strains. (PDF 278 kb)

Additional file 4: **Figure S4.** *ugtP*^*HEX-*^*-his* is expressed at the same level as WT and UgtP^HEX-^-His is stabilized in a *∆clpP* background; this file shows qRT-PCR data for *ugtP* binding mutants compared to WT *ugtP*, and also shows semi-quantitative immunoblots for UgtP-His from the previously mentioned strains cultured in both nutrient-rich and nutrient-poor media. (PDF 220 kb)

Additional file 5: **Figure S5.** A comparison of UDP-glucose molecules per cell during growth in LB and minimal sorbitol; this file shows UDP-glucose molecules per cell from WT, Δ*pgcA*, and Δ*ugtP* cells cultured in nutrient-rich media (and WT in nutrient-poor media) as measured by LC-MS/MS. (PDF 112 kb)

Additional file 6: **Figure S6.** Positive and negative controls for the ClpXP in vitro proteolysis assay; this file shows proteolysis of both a known substrate for ClpXP (Spx), and a non-targeted protein (Thioredoxin-His). (PDF 117 kb)

Additional file 7: **Figure S7.** UgtP concentration can be modulated in minimal sorbitol; this file shows semi-quantitative immunoblots for UgtP-His from strains containing either one inducible copy of *ugtP-his*, or one inducible copy and one “native” copy of *ugtP-his*, cultured in nutrient-poor media. (PDF 175 kb)

Additional file 8: **Figure S8.** Representative TLC of membrane lipids, including diglucosyl-diacylglycerol; this file shows lipid extracts from strains producing variable amounts of UgtP cultured in nutrient-poor media, separated on a TLC plate. (PDF 6249 kb)

Additional file 9: Supplemental Methods and References. Methods and references for the experiments performed in (Additional file 1: Figure S1, Additional file 2: Figure S2, Additional file 3: Figure S3, Additional file 4: Figure S4, Additional file 5: Figure S5, Additional file 6: Figure S6, Additional file 7: Figure S7, Additional file 8: Figure S8). (DOCX 23 kb)

Additional file 10: **Table S1.** Bacterial strains used in this study; this file contains a table of strains, their genotypes, and their sources used in this study. (DOCX 26 kb)

Additional file 11: **Table S2.** Oligonucleotide sequences used for qRT-PCR; this file contains a table of the oligonucleotide sequences used for qRT-PCR of various genes. (DOCX 53 kb)

## Abbreviations

DGD: Diglucosyl-diacylglycerol
LB: Lysogeny Broth
LTA: Lipoteichoic Acid
Minimal Glucose: S7_50_ + 1% Glucose
Minimal Glycerol: S7_50_ + 1% Glycerol
Minimal Sorbitol: S7_50_ + 1% Sorbitol
OPG: Osmoregulated Periplasmic Glucan
