# Supplementary figures and images for: A nutrient-dependent division antagonist is regulated post-translationally by the Clp proteases in Bacillus subtilis
Source: BMC Microbiol. 2018 Apr 6;18:29. doi: 10.1186/s12866-018-1155-2 (PMC5889556; doi:10.1186/s12866-018-1155-2)

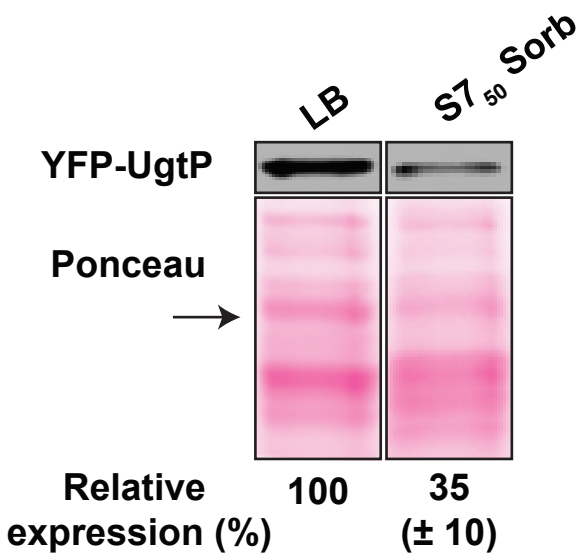

Supplement: Supplementary file 1 — Figure S1. YFP-UgtP is degraded in minimal sorbitol; this file shows ectopically expressed YFP-UgtP cultured in nutrient-rich and nutrient-poor media. (PDF 817 kb) [file 12866_2018_1155_MOESM1_ESM.pdf]

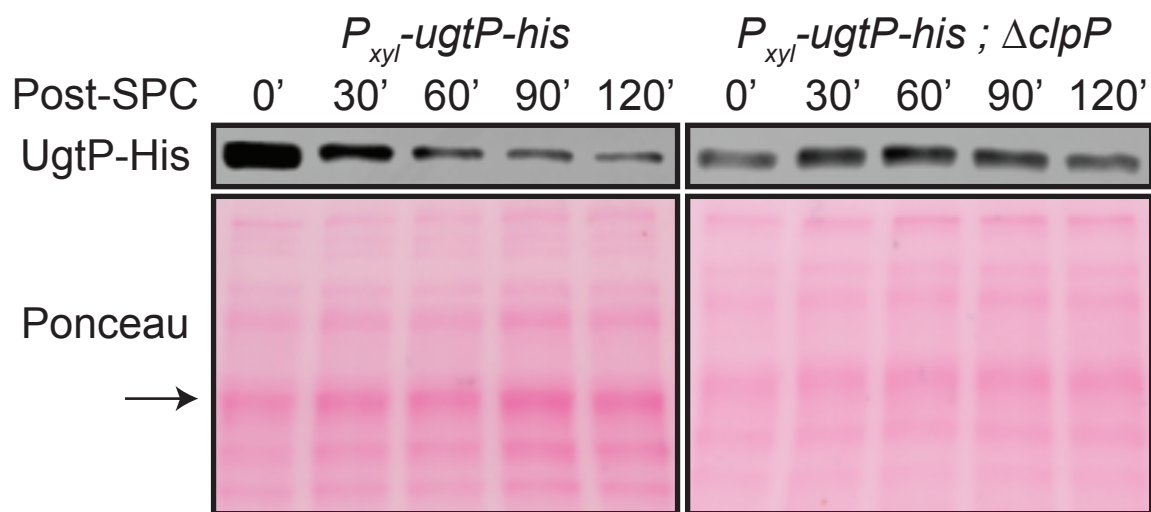

Supplement: Supplementary file 2 — Figure S2. Representative immunoblot for in vivo UgtP degradation experiment; this file shows an immunoblot of UgtP-His from cells with and without clpP, cultured in nutrient-poor media over a 2-h period, after addition of spectinomycin. (PDF 967 kb) [file 12866_2018_1155_MOESM2_ESM.pdf]

**A**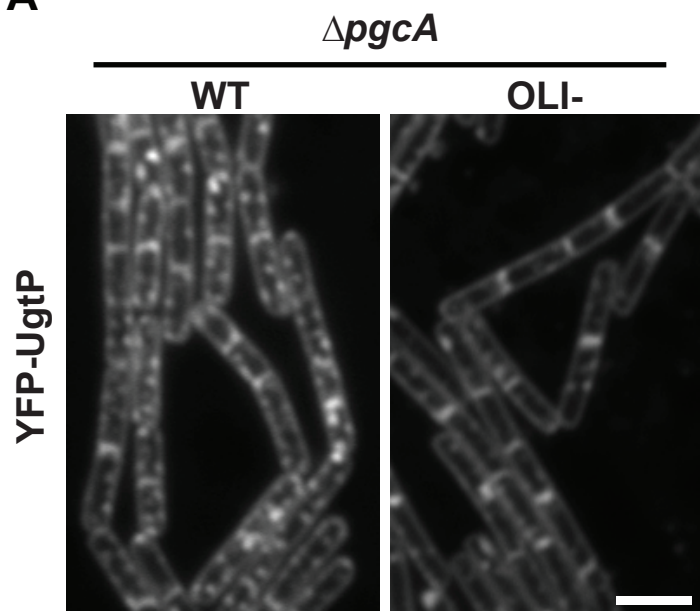**B**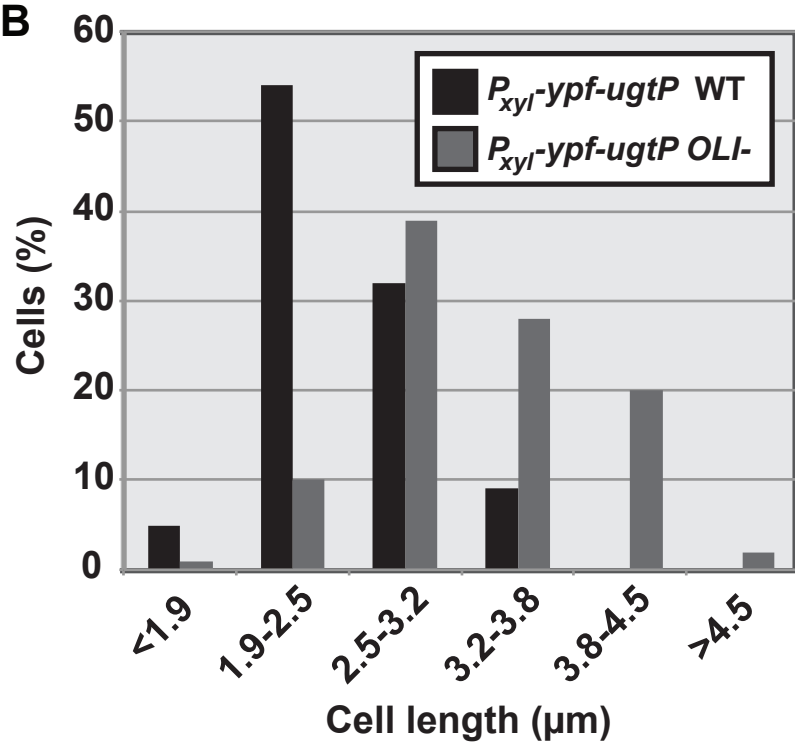

Supplement: Supplementary file 3 — Figure S3. UgtP oligomer mutant has WT localization and delays cell division in a ΔpgcA background; this file shows immunofluorescence images of cells harboring either yfp-ugtP or yfp-ugtPOLI- in a UDP-glucose null background cultured in nutrient-rich media, and also shows cell length distributions for the previously mentioned strains. (PDF 278 kb) [file 12866_2018_1155_MOESM3_ESM.pdf]

**A**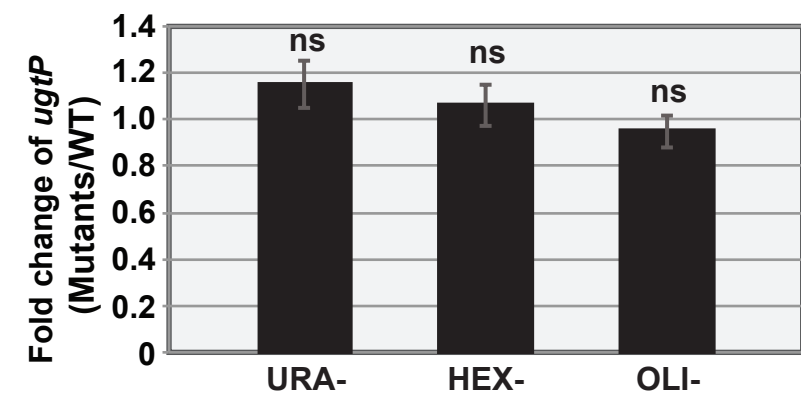**B**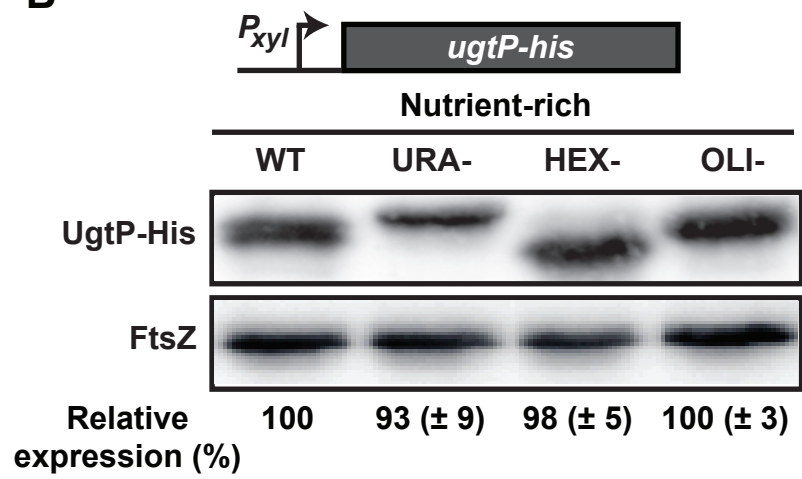**C**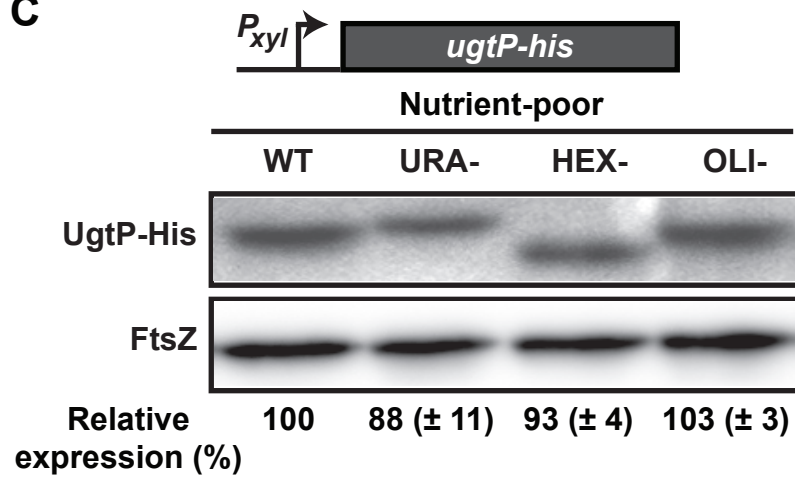

Supplement: Supplementary file 4 — Figure S4. ugtPHEX--his is expressed at the same level as WT and UgtPHEX--His is stabilized in a ∆clpP background; this file shows qRT-PCR data for ugtP binding mutants compared to WT ugtP, and also shows semi-quantitative immunoblots for UgtP-His from the previously mentioned strains cultured in both nutrient-rich and nutrient-poor media. (PDF 220 kb) [file 12866_2018_1155_MOESM4_ESM.pdf]

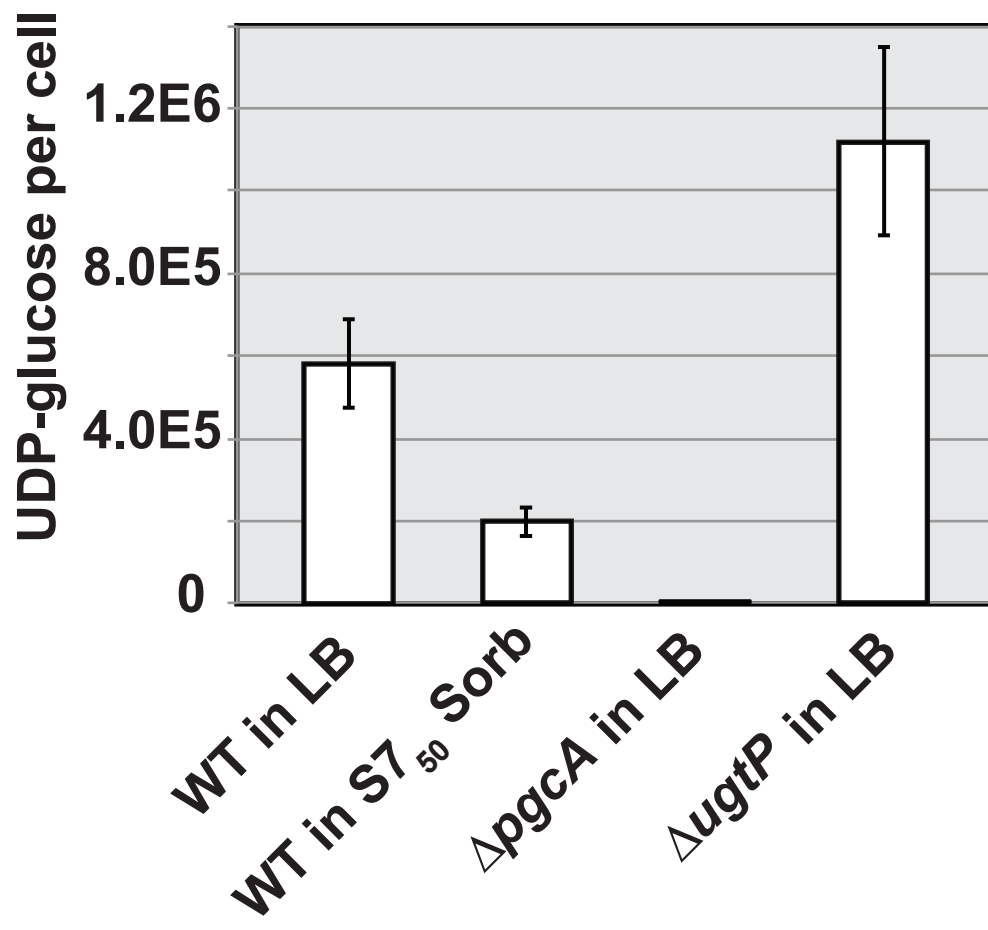

Supplement: Supplementary file 5 — Figure S5. A comparison of UDP-glucose molecules per cell during growth in LB and minimal sorbitol; this file shows UDP-glucose molecules per cell from WT, ΔpgcA, and ΔugtP cells cultured in nutrient-rich media (and WT in nutrient-poor media) as measured by LC-MS/MS. (PDF 112 kb) [file 12866_2018_1155_MOESM5_ESM.pdf]

|           |                                                                                    |     |    |     |    |     |
|-----------|------------------------------------------------------------------------------------|-----|----|-----|----|-----|
| ClpXP     | +                                                                                  | +   | +  | +   | +  | +   |
| ATP       | +                                                                                  | +   | -  | -   | +  | +   |
| Time      | 0'                                                                                 | 60' | 0' | 60' | 0' | 60' |
| Spx       | +                                                                                  | +   | +  | +   | -  | -   |
| Thio-His  | -                                                                                  | -   | -  | -   | +  | +   |
| Substrate | 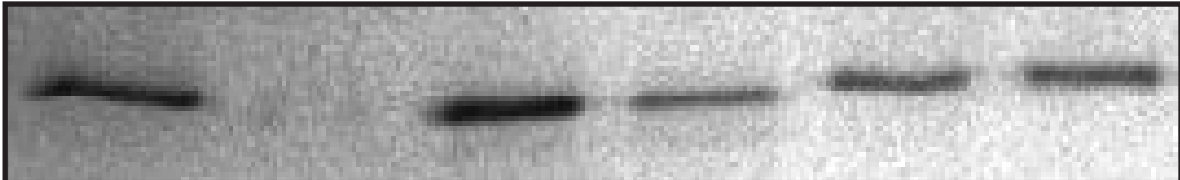 |     |    |     |    |     |

Supplement: Supplementary file 6 — Figure S6. Positive and negative controls for the ClpXP in vitro proteolysis assay; this file shows proteolysis of both a known substrate for ClpXP (Spx), and a non-targeted protein (Thioredoxin-His). (PDF 117 kb) [file 12866_2018_1155_MOESM6_ESM.pdf]

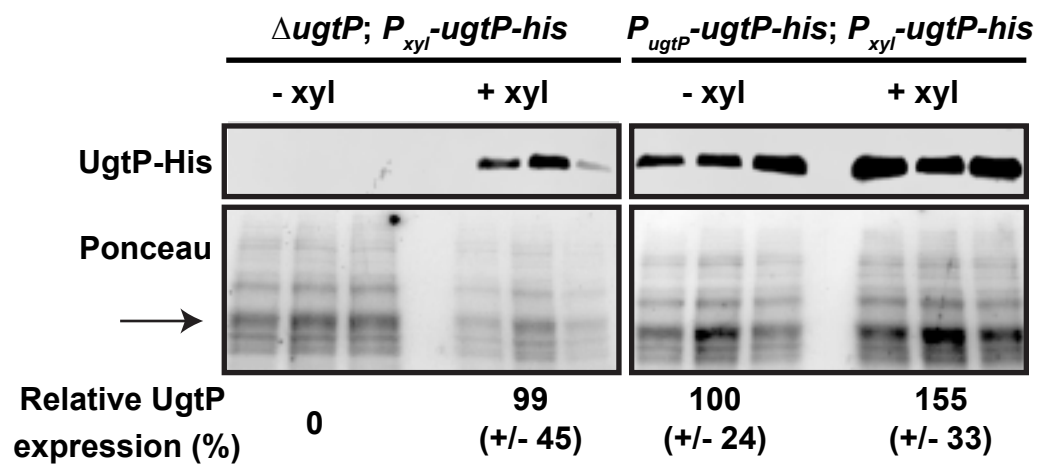

Supplement: Supplementary file 7 — Figure S7. UgtP concentration can be modulated in minimal sorbitol; this file shows semi-quantitative immunoblots for UgtP-His from strains containing either one inducible copy of ugtP-his, or one inducible copy and one “native” copy of ugtP-his, cultured in nutrient-poor media. (PDF 175 kb) [file 12866_2018_1155_MOESM7_ESM.pdf]
