# Supplementary material for: A nutrient-dependent division antagonist is regulated post-translationally by the Clp proteases in Bacillus subtilis
Source: BMC Microbiol. 2018 Apr 6;18:29. doi: 10.1186/s12866-018-1155-2 (PMC5889556; doi:10.1186/s12866-018-1155-2)
Supplement: Supplementary file 8 — Figure S8. Representative TLC of membrane lipids, including diglucosyl-diacylglycerol; this file shows lipid extracts from strains producing variable amounts of UgtP cultured in nutrient-poor media, separated on a TLC plate. (PDF 6249 kb) [file 12866_2018_1155_MOESM8_ESM.pdf]

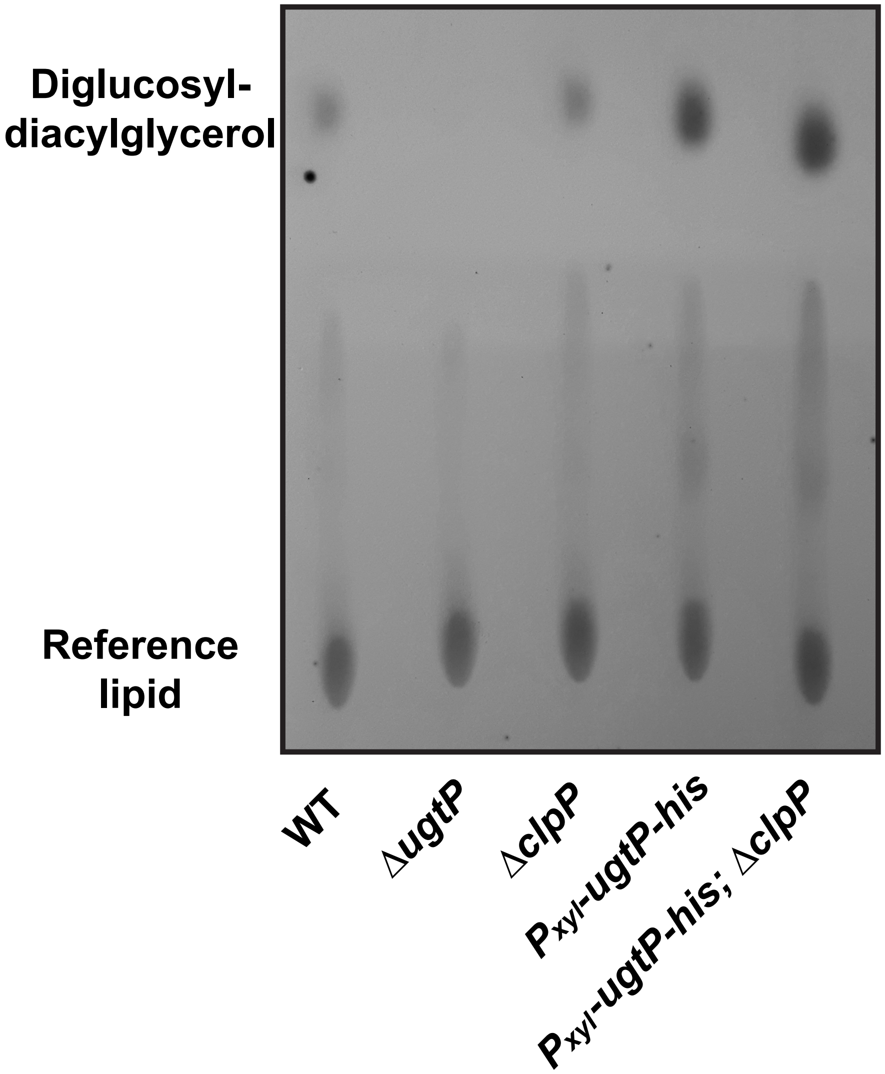
Diglucosyl-
diacylglycerol
Reference
lipid
WT
ΔugtP
ΔclpP
Pxyl-ugtP-his
Pxyl-ugtP-his; ΔclpP
