# Supplementary material for: A nutrient-dependent division antagonist is regulated post-translationally by the Clp proteases in Bacillus subtilis
Source: BMC Microbiol. 2018 Apr 6;18:29. doi: 10.1186/s12866-018-1155-2 (PMC5889556; doi:10.1186/s12866-018-1155-2)
Supplement: Supplementary file 9 — Supplemental Methods and References. Methods and references for the experiments performed in (Additional file 1: Figure S1, Additional file 2: Figure S2, Additional file 3: Figure S3, Additional file 4: Figure S4, Additional file 5: Figure S5, Additional file 6: Figure S6, Additional file 7: Figure S7, Additional file 8: Figure S8). (DOCX 23 kb) [file 12866_2018_1155_MOESM9_ESM.docx]

**Additional File 9: Supplemental Methods and References**

**Supplemental Methods**

**Strain construction**

Ampicillin was used at 100μg/ml, chloramphenicol at 5μg/ml for *B. subtilis* and 30μg/ml for *E. coli*, kanamycin at 5μg/ml for *B. subtilis* and 50μg/ml for *E. coli*, tetracycline at 12.5μg/ml, and spectinomycin at 100μg/ml. MLS resistance was selected for using erythromycin at 0.5μg/ml and lincomycin at 12.5μg/ml. Tryptophan and phenylalanine were supplemented at 40μg/ml, threonine at 80μg/ml.

The *PugtP-lacZ* transcriptional fusion was built by amplifying ~700bp of the *ugtP* promoter region using oligos: AGTCGAATTCGCAGGTTTGTATTACCATTACG and AGTCGGATCCAATGTAATCAACAACAAG. The translational fusion additionally encoded the first 90 bases of *ugtP* and was amplified with oligos: AGTCGAATTCGCAGGTTTGTATTACCATTACG and AGTCGGATCCATGTGTAACAAGTATTTCACAAAACCG. The PCR products and the vector *pDG268* were digested with *BamH*I and *Sph*I and ligated together. This construct was then transformed into PL522 facilitating the integration of *PugtP-lacZ* at the *amyE* locus.

The *Pxyl-ugtP-his* was built by amplifying *ugtP-his* from PL2265 chromosomal DNA using oligos: ATAGCATGCACATTGAGGTGAATTTACTTGAATACC and AGTCGGATCCCGATAGCACTTTGGCTTTTTG. The resulting PCR product and vector *pRCD19* were digested with *Sph*I and *BamH*I, prior to ligated together. This construct was then transformed into *B. subtilis*, which resulted in the integration of *Pxyl-ugtP-his* into the *thrC* locus.

The *his-ugtP* used in the ClpXP proteolysis assay was PCR amplified from PL522 genomic DNA using the following primers: GAGATCCATATGAATACCAATAAAAGAGTATTAATTTTGACTG and GATCGGATCCTTACGATAGCACTTTGGCTTTTTGTTTG. The *ugtP* gene product and pET28a(+) were digested with *Nde*I and *BamH*I and ligated. The resulting plasmid yielded a 6X-His tag with the linker sequence SSGLVPRGSH fused to the N-terminus of UgtP (PL3521).

The uracil, hexose, and oligomerization *ugtP* mutants were made using site-directed mutagenesis. The *Pxyl-ugtP-his*/*pRCD19* plasmid was amplified using Phusion HF DNA polymerase (NEB) with the following oligos and respective complements (not shown) (mutations underlined): uracil-binding mutant CCCGATATTATTATTAATACAGCCCCGATGATCGCCGTGCCGGAATACAG, hexose-binding mutant CCCGTGCCTGGACAGGTAAAAGAAGCAGCAAACTTCTTTGAAG oligomerization mutant GTCTTCATAAAGCTTGGGTTCACGCAAACGTGGATAAA. Following the PCR, 20U of *Dpn*I (NEB) was then added directly to the reaction and incubated at 37°C for 1h. A portion of that reaction was then transformed into AG1111 and screened by sequencing. The resulting plasmid was then transformed into *B. subtilis* and subsequently confirmed by antibiotic counter selection and threonine auxotrophy.

**Determination of UDP-glucose concentration per cell**

*B. subtilis* strains were grown to mid-log, pelleted, and extracted for UDP-glucose. GDP-glucose was added as an internal standard and used to normalize for the extraction efficiency and for quantifications. The samples were run using a LC-MS/MS using a 4000QTRAP. A standard dilution of UDP-glucose was run alongside in 3 replicates to quantify the UDP-glucose in the samples. The average nmole/g was calculated, then applied to cell counts to derive the number of UDP-glucose per cell.

**Supplemental References**

1. Perego M, Spiegelman GB, Hoch JA. Structure of the gene for the transition state regulator, *abrB*: regulator synthesis is controlled by the *spo0A* sporulation gene in *Bacillus subtilis*. Mol. Microbiol. 1988;2:689–99.

2. Weart RB, Lee AH, Chien A-C, Haeusser DP, Hill NS, Levin PA. A metabolic sensor governing cell size in bacteria. Cell. 2007;130:335–47.

3. Weart RB, Nakano S, Lane BE, Zuber P, Levin PA. The ClpX chaperone modulates assembly of the tubulin-like protein FtsZ. Mol. Microbiol. 2005;57:238–49.

4. Chien A-C, Zareh SKG, Wang YM, Levin PA. Changes in the oligomerization potential of the division inhibitor UgtP co-ordinate *Bacillus subtilis* cell size with nutrient availability. Mol. Microbiol. 2012;86:594–610.

5. Ireton K, Rudner DZ, Siranosian KJ, Grossman AD. Integration of multiple developmental signals in *Bacillus subtilis* through the Spo0A transcription factor. Gene Dev. 1993;7:283–94.

6. Nakano S, Nakano MM, Zhang Y, Leelakriangsak M, Zuber P. A regulatory protein that interferes with activator-stimulated transcription in bacteria. P Natl Acad Sci USA. 2003;100:4233–8.

7. Buske P, Levin PA. Extreme C terminus of bacterial cytoskeletal protein FtsZ plays fundamental role in assembly independent of modulatory proteins. Journal of Biological Chemistry. 2012.
