# Supplementary material for: A nutrient-dependent division antagonist is regulated post-translationally by the Clp proteases in Bacillus subtilis
Source: BMC Microbiol. 2018 Apr 6;18:29. doi: 10.1186/s12866-018-1155-2 (PMC5889556; doi:10.1186/s12866-018-1155-2)
Supplement: Supplementary file 10 — Table S1. Bacterial strains used in this study; this file contains a table of strains, their genotypes, and their sources used in this study. (DOCX 26 kb) [file 12866_2018_1155_MOESM10_ESM.docx]

**Additional File 10: Bacterial Strains Used in This Study**

| **Strain** | **Genotype** | **Source** |
| --- | --- | --- |
| *Bacillus subtilis* |  |  |
| PL522 (JH642) | *trpC2 pheAI* | [1] |
| PL2265 | PL522 *ugtP*::*ugtP-6Xhis* (Cm) | [2] |
| BW2236 | PL522 *ugtP*::*cm* | [2] |
| PL1967 | PL522 *amyE*::*PugtP-lacZ* (Cm) | This Study |
| PL2034 | PL522 *amyE*::*PugtP-ugtP90-lacZ* (Cm) | This Study |
| BH10 | PL522 *thrC*::*Pxyl-ugtP* (MLS) | This Study |
| BH12 | PL2265 *thrC*::*Pxyl-ugtP* (MLS) | [2] |
| PL2102 | PL522 *clpP*::*cm ; spx*::*neo* | [3] |
| BH129 | PL2102 *thrC*::*Pxyl-ugtP* (MLS) | This Study |
| PL2022 | BH10 *yluC*::*cm* | This Study |
| PL2028 | BH10 *cptA*::*tet* | This Study |
| PL2032 | BH10 *yvjB*::*spc* | This Study |
| PL2033 | BH10 *lonA*::*neo ; lonB*::*spc* | This Study |
| BH127 | BH10 *clpC*::*tet* | This Study |
| BH128 | BH10 *clpE*::*tet* | This Study |
| BH130 | BH10 *clpX*::*spc ; spx*::*neo* | This Study |
| BH135 | BH10 *clpC*::*spc ; clpE*::*tet* | This Study |
| BH136 | BH10 *clpC*::*tet ; clpX*::*cm ; spx*::*neo* | This Study |
| BH137 | BH10 *clpE*::*tet ; clpX*::*cm ; spx*::*neo* | This Study |
| BH138 | BH10 *clpC*::*spc* ;*clpE*::*tet ; clpX*::*cm ; spx*::*neo* | This Study |
| BH731 | PL522 *ugtP*::*tet* | This Study |
| BH736 | BH731 *thrC*::*Pxyl-ugtP* (MLS) | This Study |
| BH740 | BH731 *thrC*::*Pxyl-ugtP*∆*OLI* (I142A E146A) (MLS) | This Study |
| BH742 | BH731 *thrC*::*Pxyl-ugtP*∆*URA* (F112A V117A) (MLS) | This Study |
| BH752 | BH731 *thrC*::*Pxyl-ugtP*∆*HEX* (E306A N309A) (MLS) | This Study |
| BH767 | BH736 *clpP*::*cm ; spx*::*neo* | This Study |
| BH769 | BH742 *clpP*::*cm ; spx*::*neo* | This Study |
| BH771 | BH752 *clpP*::*cm ; spx*::*neo* | This Study |
| BH773 | BH740 *clpP*::*cm ; spx*::*neo* | This Study |
| PL1310 | *pgcA*::*cm* | [2] |
| PL2292 | *pgcA*::*spc ; amyE*::*Pxyl-yfp-ugtP* (cm) | [4] |
| JC395 | *pgcA*::*spc ; amyE*::*Pxyl-yfp-ugtP*∆*OLI* (cm) | This Study |
|  |  |  |
| *Escherichia coli* |  |  |
| PL525 | AG1111 | [5]6) |

| PL3521 | AG1111 pET28a(+) *his-ugtP* | This Study |
| --- | --- | --- |
| PL2120 | C41 (DE3) pTYB1 *clpP* | [6]3) |
| PL2118 | C41 (DE3) pTYB1 *clpX* | [6]7) |
| PL2196 | C41 (DE3) pSN17 *his-spx* | [6]7) |
| PL3370 | C41 (DE3) pBAD *thio-his* | (Invitrogen) |
| PL3141 | C41 (DE3) pET21b(+) *Plac-ftsZ* | [7] |
