# Supplementary material for: A nutrient-dependent division antagonist is regulated post-translationally by the Clp proteases in Bacillus subtilis
Source: BMC Microbiol. 2018 Apr 6;18:29. doi: 10.1186/s12866-018-1155-2 (PMC5889556; doi:10.1186/s12866-018-1155-2)
Supplement: Supplementary file 11 — Table S2. Oligonucleotide sequences used for qRT-PCR; this file contains a table of the oligonucleotide sequences used for qRT-PCR of various genes. (DOCX 53 kb) [file 12866_2018_1155_MOESM11_ESM.docx]

**Additional File 11: Oligonucleotide Sequences Used for qRT-PCR**

| **Gene name** | **Oligonucleotide sequence** |
| --- | --- |
| ***clpC*** | 5’ CTGTCGTGCTTCTTGATGAGAT |
|  | 5’ AATCGACTGTGCGTCCTTTAG |
| ***clpE*** | 5’ CTGTCGTGCTTCTTGATGAGA |
|  | 5’ AATCGACTGTGCGTCCTTTAG |
| ***clpX*** | 5’ TGCTCCGTTTCGGGTTAAT |
|  | 5’ GATCGCAACCAATGCTTCTTC |
| ***clpP*** | 5’ CAAGGTCAAGCGACAGAAATTG |
|  | 5’ GTCGCGTTCGATCACTTCA |
| ***ugtP*** | 5’ TTGGAGTGCCTGTCATTCTG |
|  | 5’ TCATCTGCAAGAAGGGAAGTG |
| ***dnaA*** | 5’ GCTCCCAATGAATTTGCCAGAGAC |
|  | 5’ GCGGTTTCGGCATAAAGTCCTCAACATC |
